# Risk Assessment of Salivary Gland Cytological Categories of the Milan System: A Retrospective Cytomorphological and Immunocytochemical Institutional Study

**DOI:** 10.5146/tjpath.2019.01469

**Published:** 2020-05-15

**Authors:** Nesreen H. Hafez, Eman S. Abusinna

**Affiliations:** Department of Pathology, National Cancer Institute, Cairo University, Cairo, Egypt

**Keywords:** Salivary glands, Fine needle aspiration cytology, Milan System for Reporting Salivary Gland Cytology, Risk of neoplasm, Risk of malignancy

## Abstract

***Objective:*** The Milan System for Reporting Salivary Gland Cytology (MSRSGC) has been recently published to help communication between cytopathologists and clinicians. The aim was to assess our institutional experience with salivary gland fine needle aspiration cytology (FNAC) and the potential applicability of the MSRSGC for the estimation of the risk of neoplasm (RON) and risk of malignancy (ROM) for each category.

***Material and Method:*** Salivary gland FNAC procedures performed at NCI, Cairo University in a three-year period from 2016 to 2018 and had a corresponding histopathological diagnosis were included in the current study. Sensitivity, specificity, positive predictive value (PPV), negative predictive value (NPV) and accuracy were estimated. Histopathological final diagnosis was the gold standard. Cytological diagnoses were re-stratified according to MSRSGC with estimation of RON and ROM for each category.

***Results:*** A total of 118 cases were included in the current work. Sensitivity, specificity, PPV, NPV and accuracy were 84.6%, 88.2%, 78.6%, 91.8% and 87%, respectively. Cytological diagnoses were re-classified as non-diagnostic (2.5%), non-neoplastic (14.4%), atypia of undetermined significance (AUS) (6.8%), benign neoplasm (40.7%), salivary gland neoplasm of uncertain malignant potential (SUMP) (7.6%), suspicious for malignancy (8.5%), and malignancy (19.5%). The RON and ROM for each category were as follows: non-diagnostic (100%, 33.3%), non-neoplastic (17.6%, 11.8%), AUS (50%, 37.5%), benign neoplasm (97.9%, 2.1%), SUMP (88.9%, 44.4%), suspicious (90%, 60%), and malignancy (100% for each).

***Conclusion:*** The Milan System for Reporting Salivary Gland Cytology is a helpful classification system. The calculated ROM for each category of the studied cases was slightly above the published MSRSGC rates but still supported the recommended management for the patient.

## INTRODUCTION

Salivary gland neoplasms represent 4% to 6.5% of all head and neck lesions. Malignancy rates of 82%, 43% and 25% have been recorded for the minor salivary gland, submandibular gland and parotid gland, respectively (1). Appropriate therapy of salivary gland tumors necessitates a precise preoperative diagnosis. Non-neoplastic lesions can be handled conservatively with medical therapy and follow-up, while neoplastic lesions require surgical intervention with major surgery for high grade malignancies (2).

The initial diagnostic workup of salivary gland lesions is based on ultrasound, computed tomography (CT) or magnetic resonance imaging (MRI) that determines the exact location of the lesion within the salivary gland and the imaging features of the nodules. They provide crucial information that aid in the surgical planning (3). However, imaging modalities failed to differentiate between benign and malignant lesions with confidence in most cases (4).

Fine‐needle aspiration cytology (FNAC) has gained a wide acceptance among clinicians for the preoperative evaluation of salivary gland lesions over incisional biopsy which has the risk of fistula formation, tumor implantation and facial nerve damage in the parotid region (5). FNAC is a quick, economical and less invasive test that is easily applied in an outpatient setting (4). It is the favored diagnostic technique to differentiate non-tumorous lesions from tumors and to identify the malignant potential of the tumors with an accuracy ranging from 81% to 98%. This rate falls to 60-75% when specific tumor subtypes are considered (6). Unnecessary surgery could be obviated in about 33% of cases based on preoperative cytological diagnosis and thus can decrease the overall management cost of salivary gland tumors (7). The accuracy of salivary gland FNAC relies on various factors such as the aspiration technique (whether with image guidance or free handed), cytological preparation (whether conventional or liquid based), intra-tumor heterogeneity and experience of the cytopathologists (2). The considerable diversity of salivary gland tumors with overlapping morphological features as well as the rarity of these tumors create a major cytological interpretation challenge in some cases (3). Therefore, cytological interpretation that is descriptive without a definitive diagnosis could confuse the clinicians in the management choices (2).

Until recently, there was no uniform reporting system for interpretation of salivary gland lesions, which made it hard for clinicians to understand the reports and led to management dilemmas (7). To create a standardized practical reporting system that aids in the communication between clinicians and cytopathologists, advance patient care, as well as allow exchange of study data between various laboratories, the American Society of Cytopathology (ASC) and the International Academy of Cytology (IAC) have suggested a classification system: The Milan System for Reporting Salivary Gland Cytology (MSRSGC) (8). This system is similar to the cytological reporting systems of the thyroid, cervix, and the pancreaticobiliary, respiratory and urinary systems (5). MSRSGC contains six diagnostic categories that are associated with proposed risk of malignancy (ROM) and recommendations for clinical intervention (2). As this system is still novel, further studies are required to determine its effectiveness and ROM of each category of the system (2,4).

The aim of the current work was to determine the cytological variety of salivary gland lesions presented to the Egyptian National Cancer Institute (NCI) over a period of 3 years, determine the diagnostic accuracy of salivary gland FNAC for distinguishing benign from malignant lesions, re-classify the salivary gland cases based on MSRSGC criteria, and define the risk of neoplasm (RON) and risk of malignancy (ROM) for each category of MSRSGC in cases which had histopathological follow-up.

## MATERIAL and METHODS

This was a retrospective three-year study (2016-2018). Review of the registry of the Cytology Unit, Pathology Department, NCI, Cairo University in this period revealed 245 cases with cytological diagnoses of salivary gland lesions. Of these, only 118 cases (48.2%) had corresponding histopathological diagnosis and these were the cases included in the current study. Unavailability of corresponding histopathological diagnosis for any case may be due to non-surgical management of non-neoplastic cases and some benign neoplasms or due to escape of some patients from therapy. Informed consents were initially obtained from all patients for the cytological and surgical procedures and for the use of tissues for research purposes following the regulations of the Ethical Committee of the National Cancer Institute.

Relevant patients’ demographic data including age, sex and the anatomical location of the lesion were recorded from patient files. Slides of the cases including cytology smear and cell block slides were retrieved from the archive of the Cytology Unit. All cytological aspirations were carried out using 23-gauge needles with an average of 2 to 3 passes depending on the size and yield of the lesion. Rapid on-site evaluation (ROSE) for the adequacy of smears was carried out at the time of aspiration using May-Grünwald Geimsa (MGG) stain on one slide. The remaining slides were immediately fixed in 95% ethyl alcohol. The retrieved smear slides were stained using modified Papanicolau stain and MGG, whereas the cell block sections were stained with H&E. In addition, immunocytochemical slides were available for some cases that required immunohistochemical verification to reach a definitive diagnosis at the time of the initial diagnosis. The slides of each case were reviewed to confirm the diagnoses and the cytological diagnoses were re-stratified according to the previously published criteria of MSRSGC (3,8) by the two authors without knowing the initial cytological interpretation or the final surgical diagnoses.

For statistical analysis, categories II (non-neoplastic), III (atypia of undetermined significance, AUS) and IVA (benign neoplasm) were combined in a negative group whereas categories IVB (salivary gland neoplasm of uncertain malignant potential, SUMP), V (suspicious for malignancy) and VI (malignancy) were combined in a positive group. This classification is based on the fact that the categories in each group have almost similar therapeutic management of their cases with minor discrepancies that might have little clinical implication.

Upon comparing cytological diagnosis with its histopathological counterpart, the cytological cases were additionally sub-classified into true positives, true negatives, false positives (interpreted inaccurately on cytology as positive and proved to be benign on excision) and false negatives (misdiagnosed cytologically as negative and turned out to be malignancy on histopathology). Sensitivity, specificity, positive predictive value (PPV), negative predictive value (NPV) and diagnostic accuracy of the cytological interpretation were estimated. Histopathological final diagnosis was considered as the gold standard.

Furthermore, the corresponding histological follow-up of cytological cases was further differentiated into non neoplastic lesions, benign neoplasm and malignant tumors. The risk of neoplasm (RON) and the risk of malignancy (ROM) for each of the six categories of the MSRSGC were calculated.

## RESULTS

This retrospective study included a total of 118 cases that underwent FNAC of salivary gland lesions during the study period and had corresponding histopathological follow-up (specimen or biopsy). Accordingly, the cyto-histopathological association could be assessed in all studied cases. One hundred two cases (86.4%) were aspirated blindly and 16 cases (13.6%) were aspirated under image guidance. The age of the studied 118 cases ranged from 6 to 86 years with a mean of 48.2±14.71. Of the studied cases, 66 (55.9%) were male and 52 (44.1%) were female. The male to female ratio was 1.3:1. The most frequently involved salivary gland was the parotid gland (109 cases, 92.4%) followed by the submandibular gland (9 cases, 7.6%). No cases with minor salivary gland affection were identified in the current work. Most cases had left salivary gland involvement (65 cases, 55.1%). Forty seven cases (39.8%) had right gland lesions. Bilateral salivary gland lesions were noticed in 6 cases making up 5.1% of the cases.

**Figure 1:**
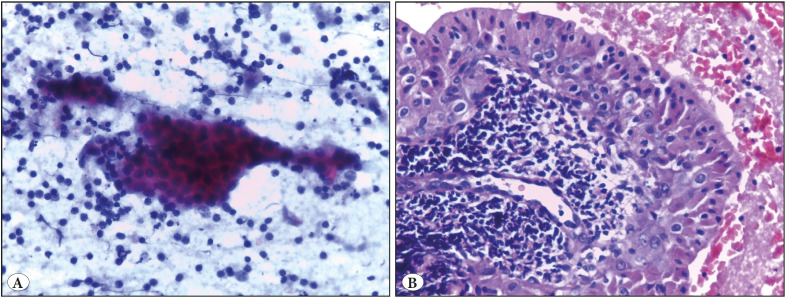
Warthin’s tumor of the parotid gland. **A)** Smear featuring monolayered sheets of oncocytic cells in a lymphoid background (Papanicolaou; x400). **B)** Cell block of the same case showing papillary structure lined by oncocytic cells with its core filled with lymphoid cells (H&E; x400).

**Figure 2:**
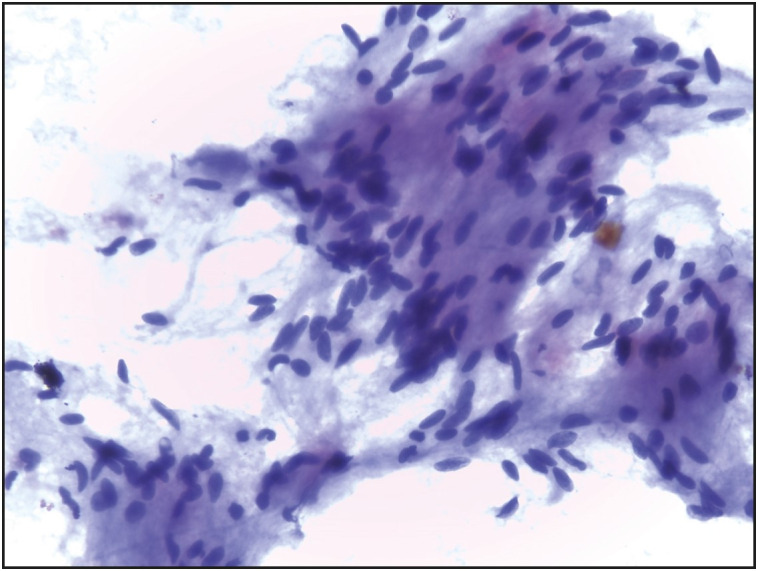
Smear from a case of schwannoma of parotid gland formed of proliferating benign-looking spindle-shaped tumor cells embedded in an afibrillary eosinophilic matrix (Papanicolaou; x400).

**Figure 3:**
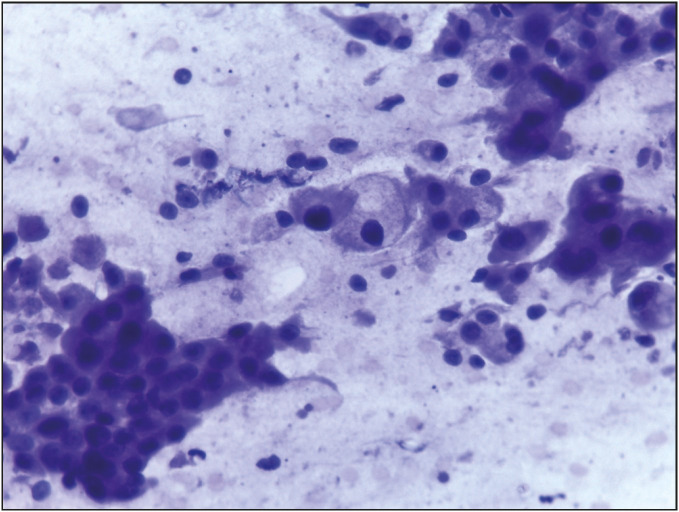
Smear from a case of mucoepidermoid carcinoma showing sheets of squamous cells with dense scanty cytoplasm and scattered glandular cells having abundant fine vacuolated cytoplasm (Papanicolaou; x400).

**Figure 4:**
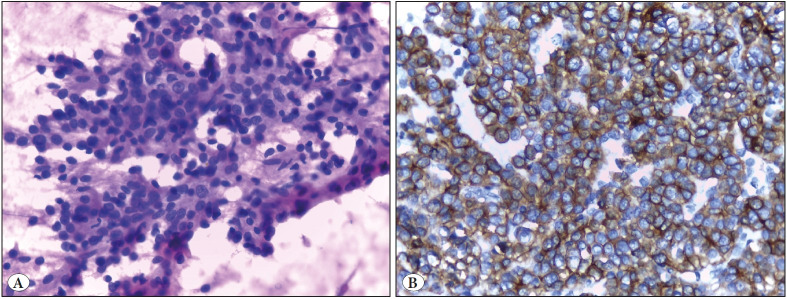
Case of acinic cell carcinoma. **A)** Smear featuring sheet of acinic cells with round nuclei and abundant granular cytoplasm (Papanicolaou; x400). **B)** Positive immunologic reaction of tumor cells to CK7 on cell block section (IHC; x400).

**Figure 5:**
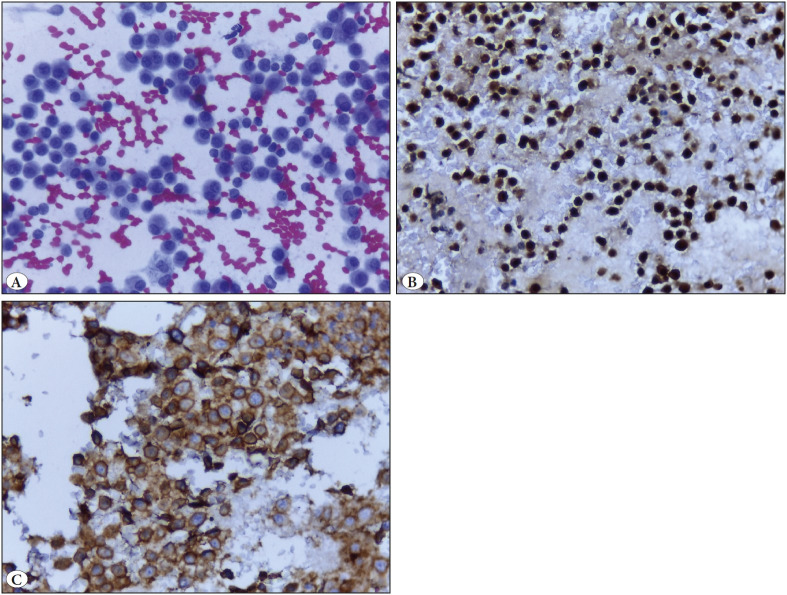
Case of plasma cell myeloma of parotid gland. **A)** Smear with dispersed plasmacytoid cells with binucleated forms (Papanicolaou; x400). **B)** Positive immunocytochemical nuclear staining of tumor cells with MUM-1 on cell block section (IHC; x400). **C)** Positive membranous immunocytochemical reaction of tumor cells to CD56 on cell block section (IHC; x400).

**Figure 6:**
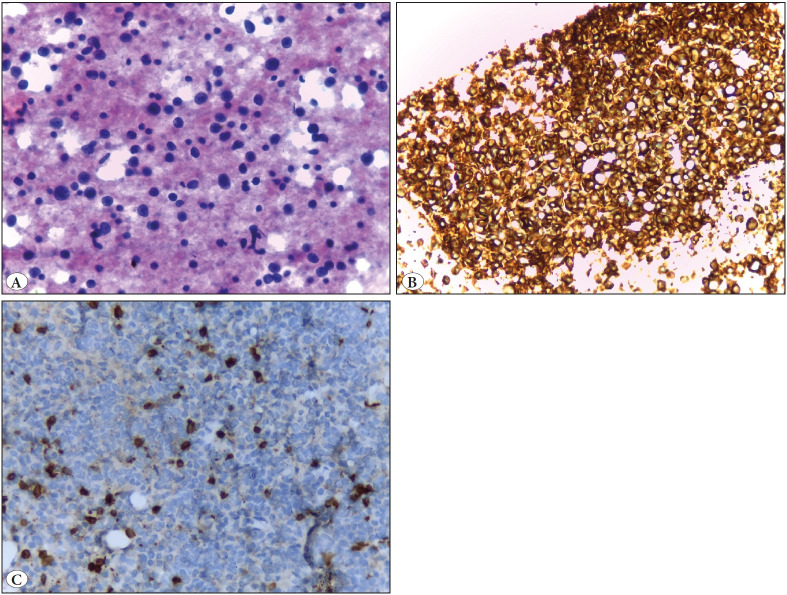
Case of non Hodgkin’s lymphoma of the parotid gland. **A)** Smear showing scattered atypical large round cells admixed with small reactive lymphocytes (Papanicolaou; x400). **B)** Cell block section showing tumor cells positively stained for CD20 (IHC; x400). **C)** Cell block with positive immunocytochemical staining of reactive small lymphocytes to CD5 with large tumor cells totally negative (IHC; x400).

Re-categorization of the salivary gland cytological diagnoses according to MSRSGC recommendations was performed. The distribution of the preoperative cytological interpretation in each diagnostic category of MSRSGC is identified in Table 1 and Table 2. Overall, the rate of the non-diagnostic category (category I) was 2.5% (3/118). Non-neoplastic category (category II) was reported in 14.4% of cases (17/118) and chronic sialadenitis represented the most common cytological diagnosis (9/17). AUS category (category III) was noted in 6.8% of cases (8/118); most of these cases had a descriptive cytology report revealing various degrees of atypical lymphoid or epithelial cells but a definite diagnosis could not be made. The benign neoplasm category (category IVA) constituted the maximum number of cases with 48/118 cases (40.7%) and the most common diagnosis was pleomorphic adenoma (26/48) followed by Warthin’s tumor (19/48) (Figure 1 Figure 2). Category IVB (SUMP**) **accounted for 9/118 cases (7.6%) where it was hard to define the exact subtype of the neoplasm with the associated list of differential diagnoses. Suspicious of malignancy (category V) was reported in 10/118 cases (8.5%), while 23/118 cases (19.5%) were identified under the malignant category (category VI) (Figure 3 Figure 4 Figure 5 Figure 6). The most common interpreted malignant tumor was mucoepidermoid carcinoma (11/23).

**Table 1:** The distribution of preoperative cytological interpretation and final histopathological follow-up according to the categories I, II, II and IVA of MSRSGC.

**Categories of Milan System**	**No.**	**Cytological interpretation**	**Histopathological follow-up**		
			**Non-neoplastic (n)**	**Benign neoplasm (n)**	**Malignancy (n)**
**Category I** **(Non-diagnostic)**	3	Inadequate (3).		Pleomorphic adenoma (1), Warthin’s tumor (1).	Metastatic papillary thyroid carcinoma (1).
**Category II** **(Non-neoplastic)**	17	Acute Sialadenitis (2).	Abscess (2).		
		Chronic sialadenitis (9).	Chronic sialadenitis (6). Reactive lymphoid hyperplasia (1), Benign salivary tissue (1).		MALT Lymphoma (1).
		Retention cyst/ benign cyst (3).	Non specific sialadenitis (1), Lymphoepithelial cyst (1).		Mucoepidermoid carcinoma (1).
		Reactive salivary lymph node (3).	Reactive follicular hyperplasia (2).	Basal cell adenoma (1).	
**Category III (AUS)**	8	Atypical epithelial cells indefinite for neoplasm (3)		Warthin’s tumor (1).	Mucoepidermoid carcinoma (1), Acinic cell carcinoma (1).
		Atypical lymphoproliferative lesion (5)	Reactive follicular hyperplasia (3), Chronic sialadenitis (1).		MALT lymphoma (1).
**Category IVA** **(benign neoplasm)**	48	Pleomorphic adenoma (26)		Pleomorphic adenoma (24), Basal cell adenoma (1).	Epithelial/myoepithelial carcinoma (1).
		Warthin’s tumor (19)	Chronic suppurative inflammation with abscess (1).	Warthin’s tumor (18).	
		Benign spindle cell neoplasm (3)		Shwannoma (1), Pleomorphic adenoma, (1), Spindle cell lipoma (1).	

**MSRSGC:** Milan System for Reporting Salivary Gland Cytopathology, **AUS:** Atypia of undetermined significance.

**Table 2:** The distribution of preoperative cytological interpretation and final histopathological follow-up according to the categories IVB, V and VI of MSRSGC.

**Categories of the Milan System**	**No.**	**Cytological interpretation**	**Histopathological follow-up**		
			**Non-neoplastic (n)**	**Non-neoplastic (n)**	**Non-neoplastic (n)**
**Category IVB (SUMP)**	9	Basaloid neoplasm (3).	Chronic sialadenitis with fibrosis and oncocytosis (1).	Basal cell adenoma (1)	Adenoid cystic carcinoma (1)
		Myoepithelial neoplasm (3).		Pleomorphic adenoma (2)	Carcinoma ex pleomorphic adenoma (1).
		Oncocytic neoplasm (2)		Oncocytic cystadenoma (1).	Mucoepidermoid carcinoma (1).
		Papillary cystic neoplasm (1)			Metastatic thyroid carcinoma (1).
**Category V (Suspicious for malignancy)**	10	Suspicious for lymphoid neoplasm (2).	Reactive lymphoid hyperplasia with lymphadenitis (1),		Diffuse large B cell non Hodgkin lymphoma (1).
		Suspicious epithelial cells with squamoid morphology (2).		Warthin’s tumor (2).	
		Suspicious epithelial cells (1).		Pleomorphic adenoma (1).	
		Extensive necrosis with suspicious cells (5).			Salivary duct carcinoma (1), Mucoepidermoid carcinoma (2), Acinic cell carcinoma (1), Atypical carcinoid (1).
**Category VI (Malignancy)**	23	Mucoepidermoid carcinoma (11).			Mucoepidermoid carcinoma (7), Adenocarcinoma NOS (2), Papillary cystadenocarcinoma (1), Salivary duct carcinoma (1).
		Adenoid cystic carcinoma (3).			Adenoid cystic carcinoma (3).
		Acinic cell carcinoma (1).			Acinic cell carcinoma (1).
		Poorly differentiated carcinoma (3).			Mucoepidermoid carcinoma (2), Metastatic amelanotic melanoma (1).
		Plasma cell myeloma (1).			Plasma cell myeloma (1).
		Non Hodgkin lymphoma (3).			Non Hodgkin lymphoma (3).
		Squamous cell carcinoma (1).			Metastatic squamous cell carcinoma (1)

**MSRSGC:** Milan System for Reporting Salivary Gland Cytopathology, **SUMP:** Salivary gland neoplasm of uncertain malignant potential.

After exclusion of non-diagnostic inadequate samples, concordance and discordance between cytologic and histopathologic diagnoses were calculated for the detection of critical cytologically diagnosed cases that required more serious and urgent therapy. Concordance was detected in 100/115 cases (87%), whereas 15/115 cases (13%) were discordant with 6 false negative cases and 9 false positive cases (Table 3). The false positive and false negative rates were 11.8% (95% confidence interval [CI] extended from 6.1%-21.2%) and 15.4% (95% CI extended from 6.9%-30.1%), respectively. Diagnostic sensitivity was 84.6% (95% CI extended from 69.9%-93.1%), whereas specificity was 88.2% (95% CI extended from 78.8%-93.9%). Positive and negative predictive values were 78.6% (95% CI; 63.9%-88.5%) and 91.8% (95% CI; 82.9%-96.5%), respectively. Diagnostic accuracy was 87% with 95% CI extended from 79.5% to 92%.

**Table 3:** Cyto-histopathological correlation of the 15 discordance cases.

**Milan system’s categories**	**Cytological diagnosis**	**Histopathological diagnosis**	**Comments**
**Category II** **(Non-neoplastic)**	Chronic sialadenitis	MALT Lymphoma (1)	FN
	Retention cyst/ benign cyst	Mucoepidermoid carcinoma (1)	FN
**Category III (AUS)**	Atypical epithelial cells indefinite for neoplasm	Mucoepidermoid carcinoma (1) Acinic cell carcinoma (1)	FN
	Atypical lymphoproliferative lesion	MALT lymphoma (1)	FN
**Category IVA** **(benign neoplasm)**	Pleomorphic adenoma	Epithelial/myoepithelial carcinoma (1)	FN
**Category IVB (SUMP)**	Basaloid neoplasm with atypical cytological features	Basal cell adenoma (1)	FP
		Chronic sialadenitis with fibrosis and oncocytosis (1)	FP
	Myoepithelial neoplasm favoring malignancy	Pleomorphic adenoma (2)	FP
	Oncocytic neoplasm with atypical cytological features	Oncocytic cystadenoma (1)	FP
**Category V (Suspicious for malignancy)**	Suspicious for lymphoid neoplasm	Reactive lymphoid hyperplasia with lymphadenitis (1)	FP
	Suspicious epithelial cells with squamoid morphology	Warthin’s tumor (2)	FP
	Suspicious epithelial cells	Pleomorphic adenoma (1)	FP

**AUS:** Atypia of undetermined significance, **SUMP:** Salivary gland neoplasm of uncertain malignant potential, **FN:** False negative,** FP:** False positive.

The corresponding histological follow-up of the studied cytological cases was further differentiated into non-neoplastic lesions (21/118 cases, 17.8%), benign neoplasm (57/118 cases, 48.3%) and malignant neoplasm (40/118 cases, 33.9%) (Table 1 and Table 2). The RON and ROM were estimated for each diagnostic category of MSRSGC (Table 4). The three cases of non-diagnostic interpretation on cytology proved to be Warthin’s tumor, pleomorphic adenoma and metastatic papillary thyroid carcinoma on histopathological follow-up (Table 1).

**Table 4:** The risk of neoplasm and risk of malignancy across categories of MSRSGC.

**Milan system categories**	**No.**	**RON**	**ROM**
I: non-diagnostic	3	100%	33.3%
II: non-neoplastic	17	17.6%	11.8%
III: AUS	8	50%	37.5%
IVA: Benign neoplasm	48	97.9%	2.1%
IVB: SUMP	9	88.9%	44.4%
V: Suspicious for malignancy	10	90%	60%
VI: Malignant neoplasm	23	100%	100%

**MSRSGC:** Milan System for Reporting Salivary Gland Cytopathology, **AUS:** Atypia of undetermined significance, **SUMP:** Salivary gland neoplasm of uncertain malignant potential, RON: Risk of neoplasm, **ROM:** Risk of malignancy.

Non diagnostic category had 100% RON and 33.3% ROM (Table 4). In the non-neoplastic category, one case was histopathologically diagnosed as basal cell adenoma and two were diagnosed as MALT lymphoma and mucoepidermoid carcinoma (Table 1). Therefore, non-neoplastic category had RON of 17.6% and ROM of 11.8% (Table 4). For the category of AUS, the RON was 50% and the ROM was 37.5% (Table 4). Cases of the cytological AUS category were diagnosed histopathologically as one benign case of Warthin’s tumor, and three malignant cases of mucoepidermoid carcinoma, acinic cell carcinoma and MALT lymphoma (Table 1). Among cases in the cytological benign neoplasm category, one case was diagnosed histopathologically as non-neoplastic and one case as malignant tumor (epithelial/myoepithelial carcinoma) (Table 1). Benign neoplasm category (IVA) revealed 97.9% RON and 2.1% ROM (Table 4). In the SUMP category, the RON was 88.9% and ROM was 44.4%**. **On resection, 4 of these SUMP cases were benign, 4 cases were malignant and one case was non-neoplastic (Table 2). The category suspicious for malignancy had RON and ROM of 90% and 60% respectively. The RON and ROM for the malignant category were 100% each.

## DISCUSSION

In an effort to standardize cytological terms and organize the therapeutic management of salivary gland lesions, the MSRSGC was proposed with detection of ROM for each diagnostic category and suggestions for management (3,9,10). In order to support the available published data of the MSRSGC and to report our institutional experience, we retrospectively assessed the accuracy of salivary gland FNAC over a three-year period, re-classified the cytological materials based on the criteria of the MSRSGC, and calculated RON and ROM for each category of MSRSGC.

In the present work, the diagnostic sensitivity, specificity, PPV and NPV of salivary gland FNAC were 84.6%, 88.2%, 78.6% and 91.8% respectively. These were in accordance with a previous work in which authors reported sensitivity, specificity, PPV and NPV of 84%, 84%, 64% and 94%, respectively (1). In the literature, overall sensitivity of salivary gland cytology in most series was between 69.1% and 98% and specificity of approximately 88% to 100% was detected (3,4). The overall diagnostic accuracy achieved in our study was 87% which fell near the lower limit of the previously published range of 86% to 98% (4).

In the current work, three cases were categorized as non-diagnostic due to inadequate cellularity. The suggested MSRSGC management for cases in this category is to repeat FNA or use clinical and radiological correlation (8). Our cases were referred to excision without FNAC repetition; probably due to suspicious clinical or radiological findings that necessitated surgical intervention. One turned out to be metastatic malignancy and two were benign tumors. The rate of the non-diagnostic category (2.5%) in our work was similar to the rate of less than 10% proposed by the MSRSGC (8). Our result was in the range of 1.1% to 7.8% that was detected in a prior review study (9). Rossi et al*. *(7) reported an allowed range of 10% to 15% for this category’s incidence. However in some reports, the rate reached up to 44% (11) or 50 % (12). The cause of these wide variations in the incidence of the non-diagnostic category between different studies might be related to the fact that the adequacy of FNA materials of the salivary gland lesions had no definite criteria and was widely related to variable quantitative and qualitative restrictions for a long period of time. Poor cellularity, non-mucinous cyst contents, needle positioning outside of the target nodule or improperly prepared and stained smears can be the reasons of non-diagnostic reports (2). Lack of clinical and radiological findings was also identified to be in this category (3). Recently, sixty cells representative of the target lesion with clinico-radiological correlation were suggested to be the key for adequacy based on MSRSGC regulations (2,8). The possible cause of the lower incidence rate in our work was related to the application of rapid on site evaluation (ROSE) of the yield to check the adequacy before discharging the cases. In this study, the non-diagnostic category had 100% RON and 33.3% ROM. A ROM of 25% was recommended by MSRSGC (8). Our ROM was much higher than that reported in another study (6.7%), but nearly similar to its RON (95.6%) (1). In a previous comparable study, a much lower RON of 64.5% and ROM of 16.1% were detected (2). Maleki et al*.,* 2019 (12) noticed a RON of 34% and ROM of 10.6%. Some authors reported a ROM of 0% as they found no malignancy on surgical follow-up of these cases (3). The probable cause of the elevated ROM in the current study might be the small number of our studied cases. This could also be attributed to the fact that our institution is considered a referral center for malignant cases in Egypt.

Regarding the non-neoplastic category of the MSRSGC, 14.4% of our studied cases were found to be non-neoplastic on FNAC. In the literature, this value ranged from 5.1% to 53.4% (1,9). Savant et al. (3) computed a lower incidence rate of 2% for cases in this category (3). Our calculated RON and ROM for the non-neoplastic category were 17.6% and 11.8%, respectively. The calculated ROM was slightly higher than the ROM of 10% proposed by the MSRSGC (8) and the ROM of 10.2% reported in a review work (9). Rohilla et al. (13), Rossi et al. (14) and Song et al. (2) reported relatively higher ROMs of 17.4%, 16%, 14.3%, respectively. A lower ROM of 7.1% was noticed in a previous study (1). The lowest ROM of 0% was reported by Savant et al*. *(3). The clinical management proposed for this category by MSRSGC was clinical follow up and radiological correlation (8). Within the non-neoplastic category, the discordant cases with major clinical discrepancy on surgical follow-up included a case of MALT lymphoma and a case of mucoepidermoid carcinoma that were misdiagnosed cytologically as chronic sialadenitis and retention/benign cyst, respectively (false negative cases). Chronic sialadenitis as well as nonspecific sialadenosis are recognized pitfalls in salivary gland cytology as an associated malignancy might be not aspirated leading to a false negative diagnosis or presence of associated reactive cellular atypia leading to an over-diagnosis with possible recommendation of unnecessary surgery (1,13,14). A review of the smear of the first false negative case revealed polymorphous lymphoid cell population, frequent epithelioid histiocytic cells and scattered epithelial cells. The features were still favoring chronic sialadenitis. It is advised to use ancillary techniques for any lymphoid-rich aspirate to rule lymphoid neoplasm out and confirm the benign nature of the lesions (9). Mucoepidermoid carcinoma is the commonest malignant tumor and one of the most problematic neoplasms in cytological interpretation (13). Recognition of mucin-secreting, intermediate and squamous cells in smears is essential for a precise diagnosis. However, all these features are not clearly present in most cases. Cyst fluid aspiration with only mucinous background with scattered lymphocytes and rare mucus cells may cause this under-diagnosis (2). On reviewing the smears of our second false negative cases, there was mucin-like material in the background with scattered chronic inflammatory cells and debris. Based on the published recommendations, the possibility of low grade mucoepidermoid carcinoma cannot be ruled out in such situation (9).

In the present study, the bulk of the salivary gland lesions (40.7%) were in the benign neoplasm category. This figure was similar to that reported in previous studies (1–3). When the benign neoplastic cytological diagnoses were correlated with the corresponding final histopathological diagnoses, the calculated RON and ROM were 97.9% and 2.1%. This estimated ROM was consistent with the suggested rate of MSRSGC (less than 5%), which recommended conservative surgery or clinical follow-up as management for cases in such category (8). Similarly, Song et al*.* (2) estimated 100% RON and 2.2% ROM. Higher ROMs were reported by Viswanathan et al. (1), Rohilla et al. (13) and Rossi et al. (14) where the estimated ROMs were 5%, 7.3% and 6%, respectively. The lowest ROM of 0.8% was noticed by Savant et al*.* (3). The cause of the relatively accurate RONs and ROMs in our study and other studies was related to the fact that the cytomorphological features of benign salivary gland tumors have been well described in the literature and are highly reproducible and also because of the fact that benign tumors are relatively common (11). These high RON and low ROM values could enable the clinicians to trust the cytological diagnosis and manage these cases confidently. In the present work, the cause of ROM in the benign neoplasm category was attributed to the presence of a case of low grade epithelial myoepithelial carcinoma on surgical follow-up that was cytologically misinterpreted as pleomorphic adenoma (false negative case). The reported ROM in other studies is caused by false negative interpretation predominantly of carcinoma ex pleomorphic adenoma (1,2) followed by low grade mucoepidermoid carcinoma (13,14), adenoid cystic carcinoma (12,14), epithelial myoepithelial carcinoma (14) and oncocytic carcinoma (13). Epithelial myoepithelial carcinoma can pose a diagnostic complexity to the cytopathologist. It shows a bimodal pattern of epithelial and myoepithelial cells. The appearance is usually dependent on the dominant cellular population (14). In our case, myoepithelial cells were the main cell population with hyaline basement material in the background giving the appearance of pleomorphic adenoma. At the same time, cellular atypia was mild.

In the present work, 19.5% of our studied cases were identified within the malignant category. An incidence rate of 13.8% was reported by others (2,12). Much lower incidence rates of 11% and 9.4% were calculated by Savent et al*.* (3) and Viswanathan et al*. *(1), respectively. The estimated RON and ROM for the malignant category in our work were 100% for each, which is relatively higher than the incidence of 90% published by the MSRSGC (8) as well as the incidence of 91.9% observed by others (9,11) and the 92.3% (1) indicated previously. A nearly similar ROM of 98.5% was documented by Song et al. (2). In the current study, there was no false positive case in this category. The possible cause might be related to the fact that the cytopathologists at our institution abide by the malignant morphological characteristic. Any case with uncertain malignant criteria was interpreted indeterminately with a descriptive report. Thus, our malignant results had high validity and reliability.

In spite of the adequacy of cytological smears, a definitive diagnosis is not possible even in experienced hands in some cases. It was noted that this indeterminate cytological interpretation accounted for more than 30% of salivary gland cytological diagnoses and fell into the MSRSGC categories of “AUS”, “SUMP” or “suspicious for malignancy” (15,16). These categories form a major problem for the clinicians.

In the current work, the AUS category was noted in 6.8% of our cases and was similar to that reported by others (3). The MSRSGC has recommended that the “AUS” category be used wisely and that more effort is needed to decrease this category to less than 10% (8). Pusztaszeri et al*. *(17), Song et al. (2) and Rossi et al. (14) reported 10%, 10.8% and 11% occurrence rates for the AUS category, respectively. Among different institutions from the USA, Europe and China, the reported frequency varies from 0.7% to 17.0% (16). A much lower incidence of 0.6% was observed in another work (11). Among our studied cases, the RON was 50% and the ROM was 37.5% for the AUS category. Our results fell in the RON range of 41.7% to 100% and the ROM range of 0% to 75.1% that were documented in a previous work done on five different institutions (16). Our ROM was higher than the ROM of 20% proposed by MSRSGC (8). A ROM of 100% was demonstrated by Rohilla et al. (13). Like others (12), we noticed that the ROM of this category was found to be between the ROMs of the non-neoplastic/benign and malignant categories, which was in accordance with the category description. The relatively high ROM rate of this category in the present study supported the management suggestion of surgery in the clinical-radiological worrisome lesions and FNAC repetition in radiological non annoying cases (3). Careful assessment of the smears and paying attention to any specific features before reporting “AUS” could reduce the frequency of this category and probably lower the RON and ROM (2). In the present study, one case was reported as “atypical epithelial cells indefinite for neoplasm” and diagnosed as mucoepidermoid carcinoma on histopathology (false negative case). The only significant cytological feature in addition to the atypical epithelial cells after meticulous re-examination of smears was the presence of small amount of mucin in the background which was not mentioned in the initial report. Some authors have suggested that smears with only mucin content should be included in this category but that one should be suspicious when atypical cells are present as well (5). Another AUS case with atypical epithelial cells that was diagnosed as Warthin’s tumor on excision also had an insignificant amount of inflammatory cells in smears. Warthin’s tumor with few or no lymphoid cells can cause diagnostic difficulties (16). Another case of AUS had atypical epithelial cells distributed in isolation and in small sheets with scant finely granulated cytoplasm; features that still made the precise interpretation of acinic cell carcinoma difficult (false negative case). Another five AUS cases were found to be reactive lymphoid hyperplasia in 3 cases, chronic sialadenitis in one case and MALT lymphoma in one case (one false negative case) on histopathological follow up, reflecting the significance of utilizing ancillary techniques like flow cytometry that helps detect the clonality of lymphoid cells in any atypical lymphoproliferative disorder (16) and reduces the rate of AUS by 50% or more (2). In the current work, the unavailability of material for cell block preparation or inadequate cell block materials prevented the use of these techniques.

The category “SUMP” includes smears that are certainly classified as neoplasm based on their cytomorphologic features, but a clear differentiation between benign and malignant cannot be made (5). Surgical resection is used to identify the invasive nature of neoplasm and verify malignancy (3). In the present work, SUMP was noted in 7.6% of cases. This was nearly similar to that reported in a previous work as 8.2% (2). An occurrence rate of 11.9% was estimated by others (3,12). Wei et al*.* (9) calculated a much lower incidence rate of 1.4%. In the current study, the estimated RON was 88.9% and ROM was 44.4%**.** Our results were similar to those reported by others where the reported RON and ROM were 93.5% and 41.9%, respectively (12). In another similar study, the SUMP category had 100% RON and 46.6% ROM (2). Our ROM was greater than the 35% proposed by MSRSGC (8) but located within the declared range in literature that extended from 24% to 50% (1,9,13). The surgery was the only line of treatment for cases in this category (8).

Regarding the category of suspicious for malignancy, 8.5% of our cases were in this category. This was superior to the incidence of 1.6% and 2.2% mentioned in two previous multi-institutional studies (9,18). Similarly, Song et al*.* (2) noticed an incidence rate of 2.7%. A rate of 3.5% was de-tected by other authors (12). The relatively higher incidence in the present study might be explained by the fact that any highly atypical or suspicious cells in the smears were reported with recommendation of excision to avoid discharging the case with a probable serious diagnosis. In the current study, the calculated RON and ROM were 90% and 60% respectively. The estimated ROM was comparable to the reported MSRSGC incidence of 60% (8). The published ROM for this category differed broadly from one institution to another with a range from 58.6% to 100% (2,9,11). This mostly reflected different institutional experiences and cytopathologists’ skills. The recommended management for this category is surgical intervention (3). Regarding the over-diagnosed cases in this category (false positive), one case was cytologically suspicious for lymphoid neoplasm and reactive lymphoid hyperplasia was documented on surgical follow-up. Reactive lymphoid hyperplasia creates a cytological challenge. Aspiration from the germinal lymphoid center could provide highly cellular smears and might yield several large lymphoid cells (centroblasts and dendritic cells) with many mitoses. This picture could raise the possibility of malignant lymphoma. Flow cytometric study allows accurate classification in such situations (5). Two cases had initial cytological interpretation of suspicious epithelial cells with squamoid morphology, but histopathologically turned out to Warthin’s tumors. Although cytological diagnosis of Warthin’s tumor is straightforward in most cases, oncocytic epithelium might undergo squamous metaplasia with a dirty background and cause an over-diagnosis (2). One pleomorphic adenoma case was over-diagnosed cytologically as having suspicious epithelial cells. Pleomorphic adenoma can be misdiagnosed as suspicious for malignancy or even as malignancy due to the common mixture of cellular and hyaline, mucoid, or myxoid matrix elements in some malignant tumors (2).

In general, the differences in the calculated ROM between the current study and others could be influenced by difference of sample sizes, patient demographics, microscopic features and heterogeneity of the included lesions, cytopathologists’ experiences and institutional practice settings. Incidence of salivary gland tumors among different geographical areas and races, where different studies were carried out, could also refer to the recorded difference in RON and ROM among different works (19). Overall, we noticed that most of our calculated ROMs were higher than the MSRSGC recommended rates; especially in the non-diagnostic, AUS, SUMP and malignant categories. These highly estimated ROMs might be due to calculation of ROM only in surgically excised cases.

In conclusion, the diagnostic sensitivity, specificity, PPV, NPV and accuracy of salivary gland FNAC were 84.6%, 88.2%, 78.6%, 91.8% and 87%, respectively. According to MSRSGC, the benign neoplasm category had the largest number of cases followed by the malignant category. Non-diagnostic cases were the lowest in our research. We noticed that most of our calculated ROM for each category was above the recommended MSRSGC rates; especially in the non-diagnostic, AUS, SUMP and malignant categories. Among the various statistically negative categories, the highest ROM was noticed in the AUS category (37.5%); supporting the management recommendation of surgery in the clinical-radiological worrisome lesions and repetition of FNAC in non annoying lesions. The high accuracy of RON and low ROM in the benign neoplasm category could allow clinicians to trust the cytological diagnosis and manage the case confidently. On the other hand, the highest ROM for positive categories was of the malignant category followed by the SUMP one; favoring the recommendation of surgical intervention rather than conservative clinical management.

The included cases in the current study were of any age and sex; however, only cases that had final corresponding histopathological diagnoses were selected for accurate estimation of RON and ROM and all other statistical analyses. This selection could be a limitation of the current work as it could influence the risk ratios.

## Conflict of Interest

The authors declare no conflict of interest.
